# Association of Abdominal Circumference with Stepping Reaction Time and Functional Balance Among Adults: A Cross-Sectional Study

**DOI:** 10.3390/medicina61112021

**Published:** 2025-11-12

**Authors:** Shaikha Jadi M. Alsaheli, Danah Alyahya, Faizan Kashoo, Rima Almutairi, Aamal Almutairi, Muhannad Aloufi, Nouf Alsahli, Saud Alsahli, Turki Alzhrani, Shagun Agarwal

**Affiliations:** 1 Department of Physical Therapy and Health Rehabilitation, College of Applied Medical Science, Majmaah University, Al Majma’ah 15341, Saudi Arabiaf.kashoo@mu.edu.sa (F.K.);; 2 College of Dentistry, Majmaah University, Al Majma’ah 15341, Saudi Arabia; 3 College of Medicine, Majmaah University, Al Majma’ah 15341, Saudi Arabia; 4 School of Allied Health Sciences, Galgotias University, Greater Noida 203201, India; shagunmpt@gmail.com

**Keywords:** balance, dominant foot, falls, obesity, stepping reaction time

## Abstract

*Background and Objectives:* Abdominal obesity significantly impacts postural control and fall risk, yet its specific association with stepping reaction time (SRT), a critical component of balance recovery, remains underexplored in obese individuals. This study investigated the relationship between abdominal circumference (AC) and SRT while considering gender and body mass index (BMI). *Materials and Methods*: Cross-sectional observational study conducted at Majmaah University Virtual Reality Laboratory using advanced motion capture technology. In this cross-sectional study, 199 adults (104 males, 89 females) underwent AC measurement and SRT assessment using the Stability and Balance Learning Environment Apparatus (STABLE) with motion capture technology. Multiple linear regression analyses were performed to identify associations between the variables. *Results*: The regression model for right SRT was statistically significant (*F*(8, 184) = 10.24, *p* < 0.001), explaining 30.8% of variance. Limits of stability with legs apart was negatively associated with right SRT (β = −0.144, *p* = 0.039), while left SRT was strongly associated (β = 0.451, *p* < 0.001). AC did not show any association with either right or left SRT. Males demonstrated significantly faster left SRT than females (b = −0.061, *p* = 0.048), and age was positively associated with left SRT (β = 0.203, *p* = 0.002). *Conclusions*: While AC shows correlation with overall obesity measures, it is not significantly associated with stepping reaction time when evaluated concurrently with balance performance and demographic factors. Limits of stability, age, and gender were more consistently associated with stepping reaction time. The cross-sectional design of this study precludes causal inference; longitudinal investigations are necessary to confirm these associations before implementing targeted fall prevention strategies.

## 1. Introduction

The increasing prevalence of obesity is a significant public health issue [1], especially abdominal obesity, which is linked to a higher risk of several chronic illnesses [2,3]. Abdominal obesity refers to excess fat deposits in the abdominal region [4]. It has become a common concern among individuals due to a sedentary lifestyle and unhealthy habits [5]. Compared to other anthropometric measures, indicators of abdominal adiposity, such as abdominal circumference (AC), may be more accurate markers of health risks such as cognitive decline which was reported to have lower than normal AC [6] and there is an increased risk of hyperuricemia associated with increased AC [7]. Specifically, AC is a simple method for evaluating abdominal fat, and it is considered normal when a man’s AC is less than 102 cm and a woman’s is less than 88 cm [8]. AC can significantly contribute to balance issues [9]. Furthermore, recent studies have shown that the prevalence of falls and balance problems is higher in individuals with a higher body mass index (BMI) and larger AC [10,11]. According to a study, obesity alters the somatosensory and mechanoreceptor systems in the feet [11], which contribute to postural instability [12]. Additionally, the abnormal accumulation of adipose tissue in specific body areas can alter body alignment, particularly when combined with low muscle mass [13]. The heightened vulnerability stems from complex biomechanical alterations whereby excess abdominal adipose tissue displaces the body’s center of mass anteriorly, fundamentally altering postural control strategies and demanding greater muscular effort to maintain equilibrium [14]. Therefore, a better understanding of balance control can help prevent falls and maintain stability. To achieve equilibrium and minimize falls, quick protective actions, including stepping movements, are required.

Reaction time (RT) refers to the time interval between the onset of a stimulus and the initiation of an individual’s response, which reflects the speed of central nervous system processing [15]. This ability depends on sensorimotor function and cognitive processes that allow an individual to perceive threats, manage them, and select the most appropriate response [16]. Many factors influence reaction time, including age, gender, brain injury, mental stress, handedness, BMI, and certain medications, all of which are associated with slower reaction times [17,18]. Typically, this skill is referred to as stepping reaction time(SRT), which is the time taken for the step response to maintain functional balance and reduce the risk of falls by realigning the body’s center of mass and base of support [19,20]. A faster and shorter reaction time are key components of this response, helping to minimize the risk of falling when an individual reacts to an unexpected stimulus [21,22]. Recent studies have provided evidence supporting the association between obesity and certain reaction time tasks, such as visual and auditory reaction times, using various obesity indices [23,24,25,26]. The findings demonstrate a relationship between these indices and slower reaction times in obese individuals compared to non-obese individuals [27]. Obese individuals, especially those with higher AC, are at increased risk of postural instability, which can lead to falls and balance disturbances [28].

Despite the known effects of obesity on reaction times and balance, the specific relationship between abdominal circumference and SRT in obese individuals, particularly across different genders and BMI groups, remains underexplored. This study aimed to examine the associations between abdominal circumference and stepping reaction time while considering the concurrent effects of gender, body mass index, age, balance performance, and other biomechanical factors. This study hypothesizes that significant associations exist between abdominal circumference and stepping reaction time, and between limits of stability and stepping reaction time in adults with varying obesity levels. Additionally, we hypothesize that demographic factors such as gender and foot dominance will be concurrently associated with differences in stepping reaction time.

## 2. Materials and Methods

A cross-sectional study was carried out between May and September 2023 at the Department of Physical Therapy, Virtual Reality Lab, College of Applied Medical Sciences, Majmaah University, Majmaah, Kingdom of Saudi Arabia. The study was approved by the Majmaah University Ethical Committee (Ethics Ref. May. 23/COM-2023/18-5) according to the Declaration of Helsinki guidelines. The nature and purpose of the study were explained to all participants before invitation for participating in the present study. Participants provided an informed consent form prior to participating. Participants were community-dwelling volunteers recruited through university-wide digital notice boards, email announcements to staff and students, and posted flyers in local community centers within a 20 km radius of the university. While this approach facilitated recruitment of a diverse sample across BMI categories, the university-affiliated recruitment strategy may have selected individuals with higher health literacy and educational attainment compared to the general population, which should be considered when interpreting generalizability of findings.

Inclusion criteria was as follows:

1. Stable body weight (±3 kg) for the preceding three months to eliminate confounding effects of recent weight fluctuations.
2. No current pharmacological treatments known to affect balance or reaction time.
3. Corrected visual acuity of 20/40.
4. Age between 18 and 55 years. The age limitation of 55 years was implemented to minimize age-related confounding effects on reaction time performance, as established normative data demonstrate significant increases in stepping reaction times beyond this age threshold [20].The dominant foot was established by asking which leg the participants were using while kicking the ball [29].
5. Ability to ambulate independently without assistive devices.

Exclusion criteria was as follows:

1. Neurological conditions defined as any diagnosed central or peripheral nervous system disorder including but not limited to stroke, multiple sclerosis, peripheral neuropathy, or traumatic brain injury with residual symptoms.
2. Previous fall history exclusion specifically referred to any unintentional ground-level fall within the preceding 24 months, documented through participant self-report and medical record verification where available.
3. Psychiatric disorder exclusions included active major depressive disorder, generalized anxiety disorder with moderate-to-severe symptom burden, or any psychotic spectrum disorder, as these conditions may influence motor performance and cognitive processing during reaction time assessments.

### 2.1. Participants Recruitment

**Figure 1:**
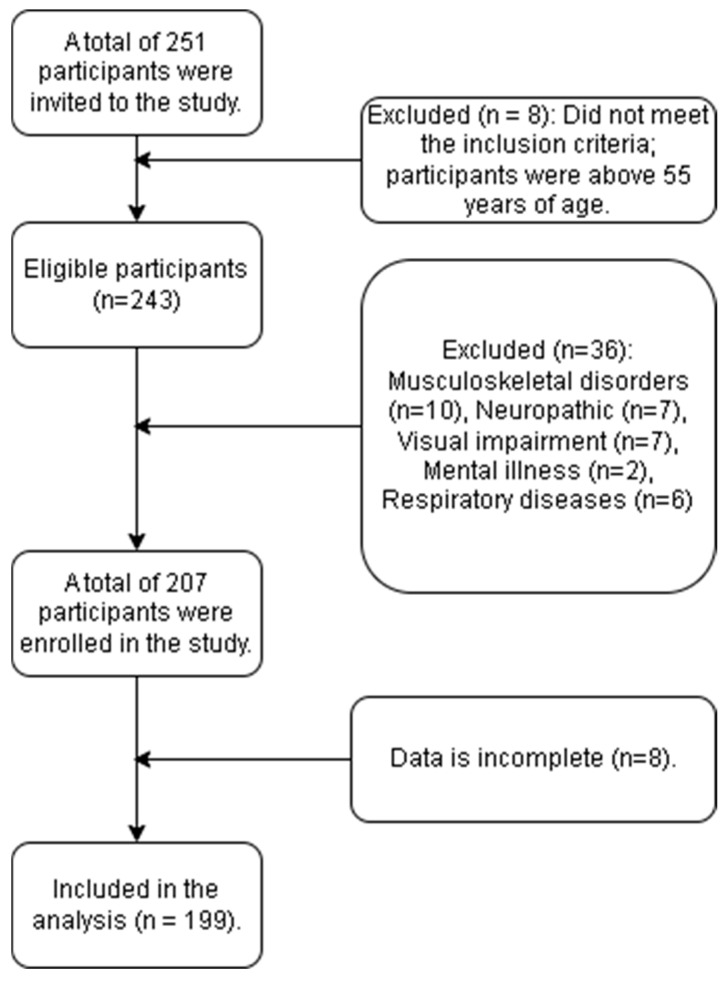
Flowchart of participant recruitment.

A total of 251 community-dwelling elderly individuals residing in Majmaah Province, Riyadh, Saudi Arabia, were initially invited to participate in the study conducted between May and September 2023. Of these, 8 individuals were excluded for not meeting the inclusion criteria, as they were above 55 years of age. The remaining 243 participants were considered eligible. Subsequently, 36 participants were excluded due to specific health conditions, including musculoskeletal disorders (n = 10), neuropathic conditions (n = 7), visual impairment (n = 7), mental illness (n = 6), and respiratory diseases (n = 6). Thus, 207 participants were enrolled in the study. However, data from 8 participants were incomplete, resulting in 199 participants included in the final analysis. The participation rate was approximately 82% (207 out of 251 invited participants). The study targeted community-dwelling elderly adults aged 55 years and below who were free from neurological, musculoskeletal, respiratory, or psychological disorders that could influence the study outcomes (Figure 1).

Participant recruitment did not employ prospective stratification by body mass index category or other demographic variables. Participants were recruited from the general community through university-wide announcements and direct outreach, resulting in a heterogeneous sample across BMI classifications. Body mass index stratification was performed post hoc during descriptive and analytical phases to characterize sample composition and facilitate sub-group comparisons. However, the lack of prospective stratification resulted in unequal group sizes across BMI categories: underweight (n = 7), normal weight (n = 42), overweight (n = 89), obese class I (n = 44), and obese class II (n = 10). This unequal distribution, particularly the small sample sizes in the underweight and obese class II categories, limited statistical power for within-group comparisons and sub-group analyses.

### 2.2. Sample Size Calculation

Sample size was calculated using the prevalence formula for cross-sectional epidemiological studies: $n=\frac{Z^{2}P(1-P)}{d^{2}}$. The calculation employed epidemiological parameters derived from Wahabi et al. (2023) [30], a comprehensive scoping review of 39 studies encompassing 640,952 participants. According to this review, the pooled prevalence of combined overweight and obesity (BMI ≥ 25) among adults aged >25 years in Saudi Arabia is 66% overall, with gender-specific variation showing 68% prevalence in males and 71% in females. Using a midpoint prevalence of 69.5% to represent the combined population, with Z = 1.96 (95% confidence interval) and d = 0.05 (±5% margin of error), the calculation yielded 326 participants. The study achieved 199 participants, representing approximately 61% of the calculated minimum, yielding an enrollment rate of 82.5% (207 of 251 invitations) and a final participation rate of 79.3% with minimal data loss of 3.9%. This pragmatic sample size provides adequate statistical precision for association analyses while demonstrating excellent recruitment performance and representativeness of the target population.

### 2.3. Anthropometric Measurements

Height measurement: Standing height was measured without shoes using a calibrated stadiometer (SECA 213, SECA GmbH & Co. KG, Hamburg, Germany), with participants positioned with heels together, back against the vertical measuring rod, and the horizontal headpiece aligned at the vertex of the skull. Body weight was measured without shoes and wearing light clothing using a calibrated digital weight scale [TANITA Body Composition Analyzer, model DC-360S, Tokyo, Japan], with participants positioned in the center of the scale platform in a standardized stance. The scale was calibrated daily using standardized weights to ensure accuracy. BMI was calculated as body weight (kg) divided by height in meters squared (kg/m^2^) and classified according to World Health Organization criteria. AC was measured using a non-elastic measuring tape (Crescent Lufkin W606PM, 60 inch/150 cm, Apex Tool Group, Sparks, MD, USA), positioned at the midpoint between the inferior border of the ribcage and the superior aspect of the anterior superior iliac spine, with the participant standing in a relaxed, upright position. The tape was positioned snugly but not constrictively, and measurement was recorded at the end of a normal expiration to standardize abdominal wall position and eliminate artificial variation from air intake. Personnel qualifications and training: All anthropometric measurements were performed by author 1 (S.M.A.) and author 4 (R.A.) who had completed standardized measurement protocols and demonstrated measurement competency prior to participant enrollment. Intra-rater and inter-rater reliability was assessed using 10 pilot participants prior to data collection, all exceeding the acceptable threshold of ICC > 0.85 for clinical research.

### 2.4. Stepping Reaction Time

**Figure 2:**
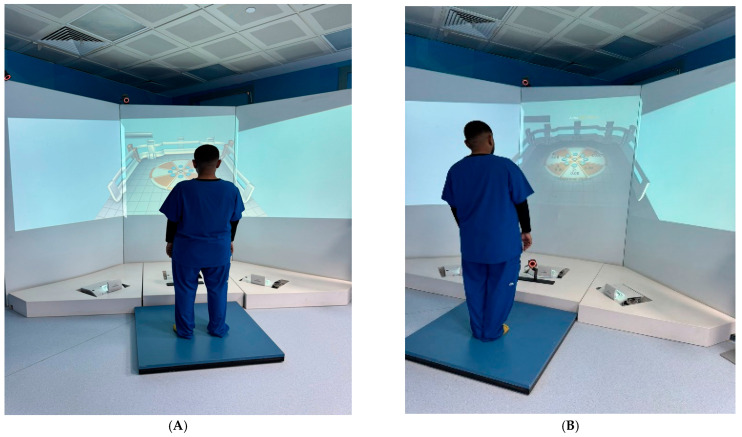
Limit of stability test: (**A**) legs apart; (**B**) legs together.

The stepping reaction time (SRT) and limit of stability (LOS) tests were conducted using the Stability and Balance Learning Environment Apparatus (STABLE) by Motek Medical (Houten, The Netherlands), which includes a 130-degree projection flat-screen virtual environment, a force plate, and four motion capture cameras to collect 3D motion data. Prior to testing, each participant was familiarized with the procedures and apparatus so that they were in a state of calmness at the time of the test. Participants were instructed to remove their shoes before beginning the tests. For the LOS Test, the participant stood in the center of the force platform with their legs together, arms by their sides, and faced the screen. Each one was instructed to move their center of pressure (COP) in eight directions as far as possible while attempting to move virtual balls on the screen without lifting their feet. This test was repeated with the participant’s legs apart. The data was recorded as the sum of displacement of virtual balls in eight directions in centimeters (Figure 2).

**Figure 3:**
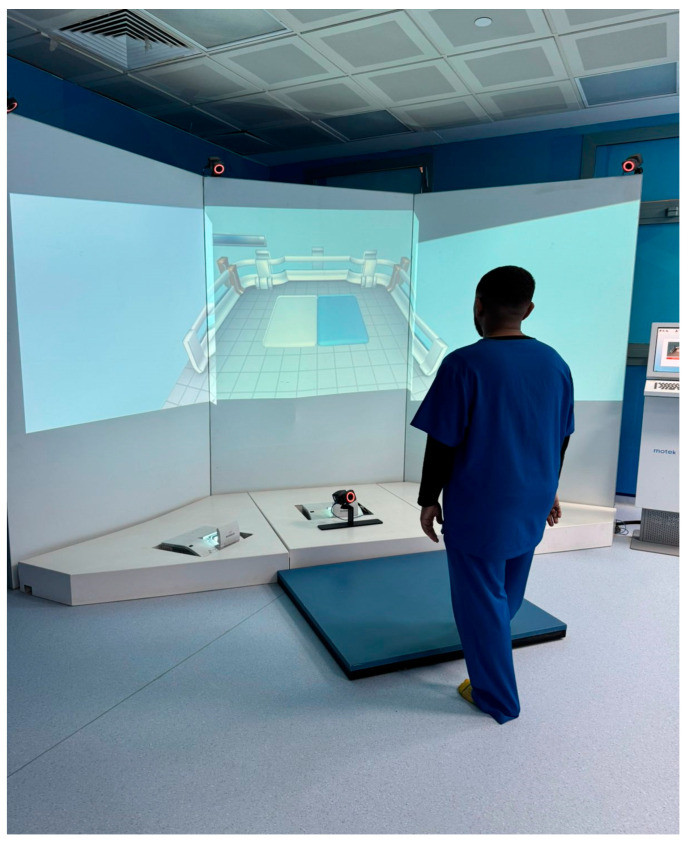
Stepping reaction time.

In the SRT test, the participant was instructed to step toward a target on the force platform in response to visual cues. When an orange panel appeared on the screen, they were asked to step with their left foot when the cue was displayed on the left side and with their right foot when it appeared on the right side. The force plate, equipped with a pressure detector, recorded the exact moment each foot made contact. Five measurements were taken for each foot, with the order of presentation randomized, and the RT for each trial was recorded in seconds (Figure 3).

The stepping reaction time assessment employed a standardized protocol developed from established clinical guidelines for reactive balance testing. Each participant received identical verbal instructions: “When you see the orange visual cue appear, step as quickly as possible toward the indicated target using the corresponding foot—left cue requires left foot stepping, right cue requires right foot stepping. Maintain your balance after each step and return to the center position.”

Visual stimuli presentation followed a randomized sequence with inter-stimulus intervals varying between 3 and 7 s to prevent anticipatory responses. The orange panel cue (dimensions: 15 cm × 20 cm) appeared for 200 milliseconds against a black background, positioned at eye level, 2.5 m from the participant. No auditory accompaniment was provided to isolate visual-motor reaction pathways specifically. Target zones on the force platform measured 30 cm × 40 cm and were positioned 45 cm lateral and 15 cm anterior to the starting position, consistent with biomechanically optimal stepping distances.

Prior to data collection, participants completed three practice trials per direction to minimize learning effects while avoiding motor pattern saturation. Each testing session comprised five measurements per foot direction, with the middle three measurements averaged to eliminate outlier effects while maintaining statistical reliability. This protocol demonstrated excellent test–retest reliability (ICC = 0.91) in preliminary validation studies conducted in our laboratory facility among 10 participants.

### 2.5. Limits of Stability Assessment Specifications

The LOS assessment utilized established protocols validated for healthy adult populations. During legs-together trials, participants maintained feet positioned parallel with medial borders touching, arms relaxed at sides. The legs-apart condition required foot placement at shoulder-width distance (measured as 1.2 × inter-acromial distance), ensuring consistent base-of-support standardization across participants.

Directional targets were presented as colored circles (diameter: 3 cm) appearing sequentially in eight directions: anterior, posterior, left, right, anterior-left, anterior-right, posterior-left, and posterior-right, each positioned at 100% of theoretical stability limits. Participants received standardized instructions to “shift your center of pressure to move the cursor toward each target as far as possible without lifting your feet or losing balance. Hold the maximum position for three seconds before returning to center.” Each directional movement was separated by 10 s rest intervals to prevent fatigue effects.

### 2.6. Data Analysis

All statistical analyses were conducted using IBM SPSS Statistics (version 20). Independent *t*-tests were employed to compare SRT by dominant foot and gender, with assumptions of normality (Shapiro–Wilk test) and homogeneity of variances (Levene’s test) verified, and Welch’s adjustment applied if variances were unequal. Descriptive statistics summarized participant characteristic with number, frequency, mean, and standard deviation. First, bivariate relationships were interrogated using Spearman’s rank-order correlation (ρ), selected for its robustness to non-normal distributions, to identify candidate predictors for multivariate modeling and to diagnose multicollinearity; this revealed a high correlation between abdominal circumference and BMI (ρ = 0.855, *p* < 0.001), necessitating their separation into distinct regression models to avoid variance inflation. Second, two separate enter-method multiple linear regression models were formulated to identify unique predictors of right and left stepping reaction time. The model for right SRT incorporated abdominal circumference, limits of stability (legs apart and together), functional reach, height, sex, foot dominance, and left SRT to control for general reactive aptitude. The model for left SRT substituted BMI for abdominal circumference to dissociate the effects of general and central adiposity, and included age, sex, foot dominance, functional reach, limits of stability, and right SRT. Both models were subjected to rigorous diagnostic checks, which confirmed that assumptions of linearity, homoscedasticity, and independence of errors were met, while variance inflation factors remained well below the threshold of 5, confirming that multicollinearity did not unduly influence the parameter estimates. This is a cross-sectional study design in which all variables are measured at a single point in time. Consequently, no temporal precedence can be established between variables, and causal mechanisms cannot be determined. All associations identified through statistical testing (correlation, regression coefficients, group comparisons) represent concurrent relationships between variables rather than causal pathways. A *p* value of < 0.05 was considered the significance level for all statistical comparisons.

## 3. Results

A total of 199 subjects participated in the study. While the study achieved adequate statistical power for the primary regression analysis, the stratified sub-group analyses by BMI category may have been limited by small cell sizes in the extreme weight categories, particularly for underweight (n = 7) and obese class 2 (n = 10) participants. Demographic data according to participant genders is outlined in (Table 1).

The sample comprised 193 participants (89 females, 104 males). Demographic variables, including age and limb dominance, were comparable between groups (all *p* > 0.05). However, significant gender differences were identified, with males demonstrating a larger abdominal circumference (*p* < 0.001), faster left hand reaction time (*p* = 0.001), and superior performance on bilateral functional reach tests (*p* < 0.05) and limits of stability measures (*p* < 0.001), indicating notable differences in body composition, psychomotor speed, and postural control (Table 1).

Table 2 presents the demographic and clinical characteristics of the study participants stratified by BMI categories. The one-way ANOVA results indicate that the groups differed significantly in age, weight, BMI, and abdominal circumference (*p* < 0.001 for all). In contrast, no significant differences were found across BMI categories for the various balance, functional reach, and reaction time measures (*p* > 0.05 for all).

**Figure 4:**
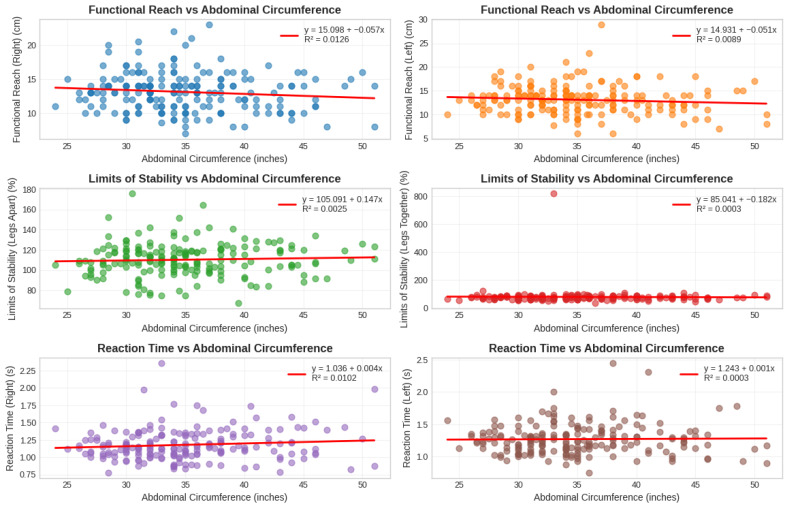
Relationship between abdominal circumference and various physical measures.

Table 3 presents the gender-stratified Spearman’s rank-order correlation analysis revealed a distinct pattern of associations between abdominal circumference and sensorimotor performance metrics. In the female cohort (n = 89), abdominal circumference exhibited statistically significant, positive correlations with both right and left reaction times (rs = 0.226, *p* = 0.033 and rs = 0.224, *p* = 0.035, respectively), while demonstrating no significant associations with functional reach or limits of stability measures (all *p* > 0.05). Conversely, in the male cohort (n = 104), abdominal circumference showed no significant correlations with any of the functional performance outcomes, including reaction time (rs = 0.061, *p* = 0.537 for right; rs = −0.061, *p* = 0.538 for left), functional reach (rs = −0.173, *p* = 0.078 for right; rs = −0.147, *p* = 0.136 for left), or limits of stability (all *p* > 0.05) (Figure 4).

A multiple linear regression was conducted to predict right foot stepping reaction time. The model was statistically significant, *F*(8, 184) = 10.24, *p* < 0.001, and explained 30.8% of the variance (Adjusted R^2^ = 0.28). Analysis of the individual predictors revealed that left foot stepping reaction time was the strongest unique contributor to the model (β = 0.451, *p* < 0.001). Furthermore, limits of stability with legs apart made a significant, unique negative contribution (β = −0.144, *p* = 0.039). No other predictors made a statistically significant unique contribution, including limits of stability with legs together (β = −0.098, *p* = 0.115), height (β = 0.119, *p* = 0.274), abdominal circumference (β = 0.079, *p* = 0.236), functional reach (β = −0.074, *p* = 0.289), foot dominance (b = −0.083, *p* = 0.101), or sex (b = −0.028, *p* = 0.545) (Table 4).

A separate multiple linear regression was performed to predict left foot stepping reaction time. This model was also statistically significant, *F*(7, 185) = 12.96, *p* < 0.001, accounting for 32.9% of the variance (Adjusted R^2^ = 0.30). The analysis revealed right foot stepping reaction time as the strongest unique predictor (β = 0.405, *p* < 0.001). Furthermore, demographic characteristics emerged as significant predictors; specifically, older age was uniquely associated with slower reaction times (β = 0.203, *p* = 0.002), and males demonstrated significantly faster reaction times than females (b = −0.061, *p*= 0.048). In contrast to the right foot model, balance performance (limits of stability with legs apart: β = −0.105, *p* = 0.127) was not a significant unique predictor, nor were BMI (β = −0.080, *p*= 0.208), functional reach (β = 0.007, *p* = 0.914), or foot dominance (b = −0.024, *p*= 0.642) (Table 5).

## 4. Discussion

This cross-sectional study investigated the association between abdominal circumference and stepping reaction time in individuals, employing advanced motion capture technology for precise measurements. The primary finding indicates that while AC shows significant bivariate correlations with age, weight, and BMI, it does not emerge as a unique predictor of SRT when considered alongside other relevant factors in multivariate models. This suggests that the relationship between abdominal obesity and balance control may be more complex than previously hypothesized and may be mediated by other variables.

### 4.1. Abdominal Circumference and Stepping Reaction Time

The finding that AC did not uniquely contribute to SRT in the regression models contrasts with some previous literature reporting significant associations between obesity measures and balance parameters [31,32]. However, our results align with research suggesting that the relationship between obesity and balance is multifactorial rather than solely determined by fat distribution. The strong correlation observed between AC and overall BMI (r = 0.855, *p* < 0.001) indicates that AC may function as a general indicator of obesity rather than specifically impacting reactive balance control through unique biomechanical pathways (Figure 4). The absence of an independent AC contribution to SRT prediction may reflect several underlying mechanisms. First, the high collinearity between AC and BMI (ρ = 0.855) suggests these measures largely capture overlapping variance related to general adiposity rather than unique biomechanical effects of central fat distribution. Second, reactive stepping performance may be more sensitive to lower extremity muscle power and neuromuscular coordination than to static anthropometric measures. The anterior displacement of center of mass caused by abdominal adiposity primarily affects static postural control, whereas rapid stepping responses depend predominantly on lower limb strength, ankle proprioception, and central processing speed, factors not directly quantified by AC measurement. Third, compensatory adaptations in obese individuals such as wider base of support during standing or altered movement strategies may mitigate the biomechanical disadvantages imposed by increased abdominal girth during dynamic tasks. These considerations suggest that AC serves as a general obesity indicator rather than a specific mechanistic determinant of reactive balance in this population.

Our findings partially support recent research distinguishing between general and central obesity in fall risk. A 2024 study found that while central obesity (measured by WC and WHtR) was significantly associated with falls and poorer performance on timed up-and-go tests, general obesity measured by body fat percentage was protective against fall-related fractures [32]. This nuanced relationship may explain why AC alone did not independently predict SRT in our sample, as its effect may be moderated by other factors such as muscle quality and distribution. To contextualize our findings, the mean SRT values observed in our sample (right foot: 1.18 ± 0.22 s; left foot: 1.27 ± 0.24 s) warrant comparison with established data. Lord and Fitzpatrick (2001) [20] reported choice stepping reaction times of 1.17 ± 0.20 s in older non-fallers aged 62–95 years, remarkably similar to our cohort’s performance despite our younger sample (mean age 25.3 years). This similarity suggests that obesity-related factors may impair reactive balance capabilities to a degree comparable to aging effects alone. Studies in younger healthy-weight adults aged 23–40 years have demonstrated faster stepping responses, with response initiation times approximately 30% shorter than those observed in our sample [33]. These comparisons underscore that the combination of obesity and increased abdominal circumference may substantially compromise stepping reaction time independent of age.

### 4.2. Balance and Demographic Predictors of SRT

The significant contribution of limits of stability (LOS) to right SRT underscores the importance of volitional balance capacity in determining reactive stepping performance. Participants with greater LOS when legs were apart demonstrated faster right SRT, suggesting that individuals with better proactive balance control may also exhibit more efficient reactive balance responses. This aligns with the concept of balance as an integrated system where voluntary and involuntary components share underlying mechanisms [31].

Age emerged as a significant predictor of left SRT, with older participants exhibiting slower reaction times. This finding is consistent with the well-established literature on age-related declines in sensorimotor processing speed and neuromuscular control [34]. The gender difference in left SRT, with males demonstrating faster reactions than females, parallels previous research on simple and choice reaction times [35]. These findings collectively highlight the importance of considering demographic factors in balance assessment and fall risk evaluation in obese populations.

### 4.3. Limb Asymmetries in Stepping Performance

The strong mutual prediction between right and left SRT in both regression models (β = 0.451 and 0.405, respectively) indicates substantial interdependence between the limbs in reactive balance control. However, the absence of a significant effect for foot dominance on SRT contrasts with some of the literature suggesting performance asymmetries in lower limb tasks [36,37]. This discrepancy may be explained by the nature of the stepping task, which potentially engages bilateral coordination mechanisms that mitigate typical dominance effects.

The finding that right SRT was generally faster than left SRT regardless of foot dominance may reflect hemispheric specialization for motor control. Research has demonstrated that the left hemisphere exhibits structural and functional advantages for motor sequencing and response execution in right-handed individuals [38]. As the majority of our participants were right foot dominant (91.7%), this neural asymmetry may have contributed to the observed right limb advantage in stepping speed.

### 4.4. Clinical Implications and Future Research

The clinical implications of our findings are noteworthy. The stronger association between SRT and LOS compared to AC suggests that balance interventions targeting volitional weight shifting and stability limits may be more effective for improving reactive stepping than focusing solely on weight reduction. Additionally, the demographic influences on SRT indicate that older and female obese individuals may require particular attention in fall prevention programs.

Future research should investigate the longitudinal relationship between AC and SRT to determine potential causal pathways. Exploring the effects of targeted interventions, such as weight loss programs combined with specific balance training, on improving SRT would be valuable. Incorporating additional measures of body composition, particularly lean muscle mass and its distribution, would help clarify the complex interplay between obesity and balance control.

## 5. Strengths and Limitations

This study is among the few to comprehensively explore the relationship between AC and SRT in individuals, employing a robust virtual reality and motion capture techniques for precise measurement. However, some limitations must be acknowledged. One of the limitations of this study is that it is a cross-sectional design, which is known to preclude consideration of cause or effect. The sample size in this study is too small to generalize the findings to a large population. The findings need to be interpreted with caution, and there is a necessity for additional statistical analyses to account for any confounding variables to draw conclusions and understand the implications of the study.

To prove this relationship, further studies in this area are needed with a larger sample size, wider age groups, and additional obesity indices such as fat % and waist-to-hip ratio. And for more reliable findings about the results of stepping reaction time, the distance between the ground and the force platforms should be considered; this might cause a delay in reaction time. Future research should investigate longitudinal changes in AC, SRT, and balance measures to better understand causal relationships. Exploring the effects of targeted interventions, such as weight loss programs and neuromuscular training, on improving SRT [39] and reducing fall risk would be valuable. Several additional methodological considerations warrant acknowledgment. First, recruitment through university advertisements may have introduced selection bias toward more educated, health-conscious individuals, potentially limiting generalizability to the broader population with obesity. Second, while our sample size was adequate for the primary regression model, stratified analyses by BMI category were constrained by small cell sizes in extreme weight classifications, precluding robust sub-group comparisons. Third, the cross-sectional design prevents inference regarding temporal relationships; longitudinal studies are needed to determine whether changes in abdominal circumference precede or follow changes in stepping reaction time. Fourth, our study did not assess body composition parameters such as muscle mass distribution or fat-free mass, which may mediate the relationship between obesity and balance control. Finally, the testing environment, while standardized, represents an artificial laboratory setting that may not fully capture real-world fall risk scenarios.

## 6. Conclusions

This cross-sectional study examined associations between AC and SRT among 199 community-dwelling adults in the Majmaah region, Kingdom of Saudi Arabia. Results indicate that AC, while correlated with overall measures of general obesity (BMI), is not significantly associated with stepping reaction time when evaluated concurrently with balance performance metrics (limits of stability) and demographic factors (age, gender). Within this sample, limits of stability (particularly the legs-apart configuration), age, and gender emerged as factors concurrently associated with stepping reaction time performance.

However, the cross-sectional design of this investigation in which all measurements were performed at a single point in time precludes definitive causal inference. The associations identified between variables represent concurrent relationships rather than established causal pathways. Although these findings may suggest potential factors relevant to balance and fall risk assessment, the evidence is insufficient to support specific clinical recommendations or fall prevention interventions until these associations are confirmed through longitudinal investigations that establish temporal relationships.

## Figures and Tables

**Table 1:** Demographic and baseline characteristics of participants by gender.

Characteristic	Female (n = 89)	Male (n = 104)	*p*-Value
**Demographic**			
Age (years), M (SD)	26.18 (8.61)	24.58 (6.21)	0.146
**Age Category, n (%)**			0.399 ^a^
Adolescent (A)	26 (29.2)	27 (26.0)	
Middle-Aged Adult (MAA)	4 (4.5)	1 (1.0)	
Young Adult (YA)	59 (66.3)	76 (73.1)	
**Limb Dominance, n (%)**			0.463 ^b^
Right	83 (93.3)	94 (90.4)	
Left	6 (6.7)	10 (9.6)	
**Anthropometric**			
Abdominal Circumference (inches), M (SD)	33.54 (5.06)	36.23 (5.76)	<0.001 *
**Neurophysiological**			
Stepping Reaction Time Rt (s), M (SD)	1.21 (0.22)	1.15 (0.22)	0.078
Stepping Reaction Time Lt (s), M (SD)	1.33 (0.26)	1.22 (0.21)	0.001 *
**Postural Control**			
Functional Reach Test Rt (cm), M (SD)	12.50 (2.25)	13.62 (3.18)	0.005 *
Functional Reach Test Lt (cm), M (SD)	12.57 (2.27)	13.64 (3.49)	0.012 *
Limits of Stability: Legs Apart (cm), M (SD)	104.08 (17.38)	115.48 (13.20)	<0.001 *
Limits of Stability: Legs Together (cm), M (SD)	78.59 (80.54)	78.73 (9.91)	<0.001 *

Note: Data are presented as mean (standard deviation) or n (%); M = mean, SD = standard deviation, Rt = right, Lt = left; *p*-values for continuous variables were derived from independent sample *t*-tests, while categorical variables were analyzed using Fisher’s exact test (denoted by superscript a) or Pearson’s chi-square test (denoted by superscript b); a significant level of α < 0.05 was applied, with asterisks (*) indicating statistically significant group differences.

**Table 2:** Participant characteristics by BMI category.

Characteristic	Total (N = 193)	Underweight (UW) (n = 7)	Normal Weight (NW) (n = 82)	Overweight (OW) (n = 56)	Obese Class 1 (OC1) (n = 38)	Obese Class 2 (OC2) (n = 10)	*p*-Value
**Demographics**							
Age (years)	25.3 (7.4)	22.1 (1.5)	22.9 (5.3)	26.8 (7.8)	28.2 (9.5)	27.6 (8.3)	<0.001
**Sex, n (%)**							0.245 ^b^
Female	89 (46.1)	1 (14.3)	36 (43.9)	27 (48.2)	18 (47.4)	7 (70.0)	
Male	104 (53.9)	6 (85.7)	46 (56.1)	29 (51.8)	20 (52.6)	3 (30.0)	
**Anthropometrics**							
Height (cm)	165.0 (9.6)	168.3 (9.2)	165.0 (9.6)	165.0 (9.2)	165.1 (10.9)	162.0 (8.4)	0.778
Weight (kg)	72.1 (17.1)	49.6 (5.0)	60.6 (9.4)	74.0 (8.8)	91.9 (14.2)	96.0 (13.1)	<0.001
BMI (kg/m^2^)	26.4 (5.5)	17.4 (0.7)	22.1 (1.9)	27.2 (1.6)	33.7 (3.0)	36.4 (1.7)	<0.001
Abdominal Circumference (inches)	35.0 (5.6)	28.9 (1.7)	31.0 (2.9)	35.8 (3.2)	41.6 (4.9)	42.1 (4.1)	<0.001
**Balance and Functional Measures**							
Functional Reach Right (cm)	13.1 (2.8)	13.6 (2.8)	13.6 (3.1)	12.9 (2.6)	12.5 (2.6)	11.6 (2.4)	0.103
Functional Reach Left (cm)	13.1 (3.0)	14.9 (3.0)	13.4 (3.0)	13.1 (3.1)	12.8 (2.9)	11.9 (2.8)	0.311
Limits of Stability: Legs Apart (cm)	110.2 (16.3)	107.9 (13.0)	110.7 (16.6)	111.5 (17.2)	107.7 (15.4)	110.1 (14.3)	0.84
Limits of Stability: Legs Together (cm)	78.7 (55.0)	74.3 (11.1)	84.9 (83.0)	74.4 (12.5)	73.4 (12.2)	74.3 (14.9)	0.766
**Reaction Time (s)**							
Right Hand	1.18 (0.22)	1.14 (0.21)	1.15 (0.23)	1.19 (0.21)	1.21 (0.21)	1.25 (0.29)	0.57
Left Hand	1.27 (0.24)	1.23 (0.21)	1.24 (0.22)	1.30 (0.23)	1.30 (0.30)	1.21 (0.15)	0.473
**Categorical Variable**							
**Dominant Foot, n (%)**							0.755 ^f^
Right	177 (91.7)	7 (100)	75 (91.5)	51 (91.1)	34 (89.5)	10 (100)	
Left	16 (8.3)	0 (0)	7 (8.5)	5 (8.9)	4 (10.5)	0 (0)	

Notes: Data are presented as mean (standard deviation) for continuous variables and n (%) for categorical variables. BMI categories: UW (underweight), NW (normal weight), OW (overweight), OC1 (obese class 1), OC2 (obese class 2). *p*-value: For continuous variables, *p*-value from one-way ANOVA. Bolded values indicate statistical significance (*p* < 0.05). ^b^ Chi-square test; ^f^ Fisher’s exact test (used due to low expected cell counts in some BMI categories).

**Table 3:** Spearman’s rank correlation matrix for study variables.

Variable	1	2	3	4	5	6	7	8
1. Age	—							
2. Height	−0.044	—						
3. Weight	0.225 **	0.444 ***	—					
4. BMI	0.275 ***	−0.016	0.866 ***	—				
5. Abdominal Circumference	0.341 ***	0.276 ***	0.894 ***	0.855 ***	—			
6. Functional Reach (Rt)	−0.202 **	0.323 ***	0.022	−0.13	−0.101	—		
7. Limits of Stability (Apart)	−0.085	0.361 ***	0.145 *	−0.007	0.061	0.295 ***	—	
8. Reaction Time (Rt)	0.182 *	−0.059	0.041	0.096	0.093	−0.088	−0.277 *	—
9. Reaction Time (Lt)	0.244 *	−0.163 *	−0.066	0.01	0.01	−0.117	−0.325 *	0.567 *

Note: The correlation matrix presents Spearman’s rho coefficients for a select subset of variables to ensure clarity and relevance to the primary research aims. Variables were included based on theoretical relevance to the outcome measures of balance and reaction time, and the strength of their bivariate correlations (*p* < 0.10). Several anthropometric variables were highly correlated (r > 0.85) with height and were excluded to prevent redundancy and multicollinearity in subsequent analyses. Correlation coefficients are indicated as follows: * *p* < 0.05, ** *p* < 0.01, *** *p* < 0.001. The (—) symbol indicates the correlation of a variable with itself. The primary purpose of this analysis was to identify potential predictor variables for inclusion in regression models, with a specific focus on factors associated with reaction time (right) and reaction time (left).

**Table 4:** Multiple linear regression predicting right stepping reaction time.

Predictor	B	SE	β	t	*p*
**(Intercept)**	0.415	0.39		1.065	0.288
**Limits of Stability (Legs Apart)**	−0.002	0.001	−0.144	−2.084	0.039
**Reaction Time (Left)**	0.429	0.062	0.451	6.879	<0.001
**Limits of Stability (Legs Together)**	−0.0004	0.0003	−0.098	−1.585	0.115
**Height**	0.003	0.003	0.119	1.097	0.274
**Abdominal Circumference**	0.003	0.003	0.079	1.19	0.236
**Dominant Foot (Left)**	−0.083	0.05		−1.649	0.101
**Sex (Male)**	−0.028	0.047		−0.606	0.545
**Functional Reach (Right)**	−0.006	0.005	−0.074	−1.064	0.289

**Table 5:** Multiple Linear Regression Predicting Left Foot Stepping Reaction Time.

Predictor	B	SE	β	t	*p*
**(Intercept)**	0.889	0.17		5.237	<0.001
**Limits of Stability (Legs Apart)**	−0.002	0.001	−0.105	−1.535	0.127
**BMI**	−0.003	0.003	−0.08	−1.264	0.208
**Age**	0.006	0.002	0.203	3.114	0.002
**Functional Reach (Left)**	0.001	0.005	0.007	0.108	0.914
**Right Foot Stepping Reaction Time**	0.425	0.069	0.405	6.145	<0.001
**Foot Dominance (Left)**	−0.024	0.053		−0.465	0.642
**Sex (Male)**	−0.061	0.031		−1.992	0.048

## Data Availability

Data will be available upon request.

## Abbreviations

The following abbreviations are used in this manuscript:

SRT	Stepping reaction time
AC	Abdominal circumference
LOS	Limits of stability
BMI	Body mass index
RT	Reaction time
STABLE	Stability and Balance Learning Environment Apparatus
COP	Center of pressure
